# The arousal paradox in critical task performance in automated driving during sleep inertia using a quasi experimental approach

**DOI:** 10.1038/s41598-025-08726-4

**Published:** 2025-06-27

**Authors:** Markus Tomzig, Johanna Wörle, Alexandra Neukum, Martin Baumann

**Affiliations:** 1 Ulm University, Albert-Einstein-Allee 41, 89081 Ulm, Germany; 2 Wuerzburg Institute for Traffic Sciences, WIVW GmbH, Robert-Bosch-Straße 4, 97209 Veitshöchheim, Germany; 3 Singapore-ETH Centre, 1 Create Way, CREATE Tower, 138602 Singapore, Singapore

**Keywords:** Sleep, Arousal, Sleep inertia, Takeover, Electroencephalography (EEG), Human behaviour, Engineering, Electrocardiography - EKG, Electroencephalography - EEG

## Abstract

Sleep inertia is the post-awakening transitional state of lowered arousal, characterized by increased low-frequency activity in the electroencephalogram (EEG) and impaired cognition. While some theories consider arousal holistically, recent research questions whether these findings apply to situations requiring immediate critical action post-awakening, such as for pilots, emergency responders, or future drivers of automated vehicles. This study compared self-reported, cortical, and physiological arousal in such a scenario. Twenty-four participants completed four drives in a driving simulator. In three drives, participants were instructed to sleep for 20, 40, and 60 min during automated driving before being prompted to resume control. The sleep stage prior to the takeover request served as a quasi-experimental independent variable. Regression analyses showed that cortical arousal was low following awakenings from N2 or N3, indicated by increased delta, theta, and alpha activity. However, beta activity and heart rate also increased, suggesting elevated physiological arousal. Significant positive correlations were found between delta activity, heart rate and self-reported sleepiness. This “arousal paradox” is not in line with the idea of arousal as a holistic concept. We hypothesize that the heightened physiological response under sleep inertia may be attributed to stress in demanding situations under sleep inertia. We conclude that forced awakenings from N2 or N3 should be avoided. If someone is nevertheless awakened from N2 or N3, they should be given sufficient time between awakening and taking over duties for arousal to normalize.

## Introduction

Awakening from sleep is characterized by sleep inertia, a transitional period marked by cognitive impairment, self-reported sleepiness, and low arousal^1,2^. Terms such as alertness or activation are used almost interchangeably with arousal^3,4^. There are various arousal theories and models, such as the Yerkes-Dodson-Law^5^the Two-Process Model of alertness^6^ as well as Duffy’s^3^ and Malmo’s^4^ arousal theories, which all have a slightly different concept of arousal. A common feature of all theories is that arousal is considered a continuous state from the lowest levels during sleep to the highest levels during excitement, anxiety, or stress. Some theories consider arousal a purely cortical state^4,7^. These theories characterize low arousal by a predominance of low electroencephalogram (EEG) frequencies (i.e., delta- and theta-waves with frequencies from 0.5 to 4.0 Hz and 4.0 to 8.0 Hz respectively^8^). Under relaxed wakefulness, alpha patterns (8.0–12.0 Hz^8^) are dominant, while states of high arousal are characterized by high-frequency beta waves (12.0–35.0 Hz)^4,8^. Other theories consider arousal a holistic state of the human organism, comprising cortical, physiological, and self-reported arousal^3^.

EEG spectral analyses of the awakening brain suggest that the transition from sleep to wakefulness is marked by a decrease in delta and theta power and an increase in alpha power^9^. Compared to pre-sleep wakefulness, however, spectral analyses contain higher delta power and lower beta power after awakening, particularly in the posterior scalp locations^10–12^ with less consistent findings for higher theta and alpha power^10,13^. These observations indicate residual sleep characteristics in the wake EEG. Along with the finding that under sleep inertia individuals experience feelings similar to sleepiness, these findings contribute to considering sleep inertia a state of low arousal^1,13^. A common explanation for performance impairments observed upon awakening is that low arousal slows cognitive processing^1,14^.

While measuring arousal as a purely cortical state is relatively straightforward, standardized parameters for arousal as a holistic state of the human organism are lacking^3^. The basic assumption is that while cortical, physiological, and subjectively experienced arousal are different dimensions of the same construct, they are all mediated by the same mechanism(s). For example, subjective sleepiness, body temperature, and task performance are closely correlated over the course of the day^15^. Subjective sleepiness over the course of the day is related to self-reported performance decrements, for example, reduced fitness to drive^16^. However, sleep deprivation and sleep inertia, both states of low arousal, may have differential effects on cognitive performance: Under sleep inertia, task speed is more impaired than accuracy, while under sleep deprivation the opposite is true^17^.

Cognitive impairments and level of arousal under sleep inertia depend on the circadian timing of awakening, prior sleep history, and the sleep stage the individual awakens from^1,13^. Sleep consists of repeating cycles in which different stages alternate, including Stage 1 (N1), Stage 2 (N2), Stage 3 (N3), and Rapid Eye Movement sleep (REM), with brief wakefulness phases (W) in between^18^. N1, N2, and N3 are collectively referred to as Non-Rapid Eye Movement (NREM) sleep, in contrast to REM sleep. Each sleep stage is characterized by distinct EEG patterns:

- **N1** is the shallowest sleep stage, marked by theta waves (4–7 Hz) and a reduction of alpha waves (8–12 Hz) compared to a state of wakefulness (W). N1 makes up about 2–5% of total sleep time^19^.
- **N2** is deeper than N1 and is characterized by the occurrence of sleep spindles (12–14 Hz) and K-complexes, and constitutes about 45–55% of sleep time^19^.
- **N3** shows predominantly delta waves (0.5–4 Hz) and accounts for 13–23% of sleep time, providing significant restorative functions^18–21^.
- **REM** sleep is associated with vivid dreaming and heightened brain activity, featuring low-amplitude, mixed-frequency EEG waves and sawtooth waves. It comprises about 20–25% of sleep time, with longer periods in later cycles. Physiological changes during REM include rapid eye movements and near-total muscle paralysis. Note that dreaming can also occur in other sleep stages^19^.

A typical night includes 4–6 sleep cycles, each lasting 90–120 min, progressing through NREM stages followed by REM sleep. Early cycles contain more N3, while later cycles have longer REM periods^19,22^. Previous research observed the most pronounced effects of sleep inertia after awakenings from N3 and – to a lesser extent – after N2^1,2,13,23^. Awakenings from N1 are commonly associated with less sleep inertia^1^. Findings on REM-awakenings are mixed, depending on whether the individual has been awakened from a phasic or tonic REM sleep substage^1,24^. Forced awakenings are known to produce higher levels of sleep inertia compared to self-awakenings^25,26^.

During NREM sleep, parasympathetic activity increases while cardiac sympathetic activity decreases^27,28^. Consequently, heart rate and blood pressure decrease, and muscle sympathetic nerve activity drops compared to wakefulness^27,29^. In contrast, REM sleep is characterized by autonomic instability where fluctuations between parasympathetic and sympathetic influences produce sudden changes in heart rate and blood pressure. Heart rate and blood pressure are higher during REM sleep compared to non-REM sleep stages^28^. Electrocortical arousal events, whether spontaneous or triggered by external stimuli, are linked to sympathetic neural surges. This results in temporary increases in heart rate, blood pressure, and muscle sympathetic nerve activity^28^. Arousal events and awakenings are typically accompanied by sharp increases in heart rate^30^. However, heart rate increases already before cortical arousals in self-awakenings^28,31^. In forced awakenings, there is no possibility for this kind of “physiological preparation” of the awakening. Hence, the physiological activation begins with the awakening stimulus^31^.

The physiological responses to self-awakenings compared to forced awakenings indicate that the context in which awakening occurs significantly impacts the response. Many studies on awakenings and sleep inertia effects are conducted in sleep laboratory settings, where participants are awakened in a calm environment while lying in bed^32^. Additionally, for justified methodological reasons, many studies start measuring sleep inertia after a delay (e.g., 90 seconds^33^ or several minutes^32^) and aggregate the measurement of sleep inertia over a relatively large period of time (e.g., 5–10 min)^10–12,32^. However, in real-world scenarios, individuals often wake up in more demanding circumstances, such as healthcare workers in hospitals, firefighters performing critical tasks, pilots resuming control of an aircraft instantly after a nap^32^ or drivers taking over control after a nap during an automated drive as the vehicle encounters a system limit^23^. These scenarios typically involve forced awakenings and require immediate action. They are characterized by high temporal demands and the need for fast decision-making and physical activity. The time period between awakening and executing (often safety-critical) tasks varies in the mentioned examples: While aviation guidelines recommend that pilots do not return to duty for 15–20 min after a nap^34,35^ real-life examples show that pilots do not always adhere to these guidelines and return to duty immediately after awakening. An example is the incident report of a 2011 Air Canada flight where sleep inertia was identified as the main cause of an incident with seven injured passengers: The first officer had returned to duty less than 1 min after awakening from an on-board nap^36^. In automated driving scenarios, drivers might need to take action immediately in the moment of awakening. Examples include the illicit napping of Tesla’s Autopilot users^37,38^ or misuse of higher automated driving systems^39^. A unique challenge in the automated driving scenario is that, upon awakening, the driver must immediately take control of the vehicle without the opportunity to engage in any preparatory physical activity^40^. Forced awakenings produce higher performance impairments compared to self-awakenings^25,26^. Besides that, it is not clear how other contextual factors impact arousal upon awakening: In a driving simulator study on awakenings during an automated drive, mean heart rate as a measure of physiological arousal was increased throughout 10 min after awakening to a takeover request compared to a condition where participants did not sleep. At the same time, participants reported subjectively lower arousal after awakening from sleep compared to the wakefulness condition^41^. This finding is inconsistent with the idea of a holistic arousal concept^3^. In another study on awakenings to a takeover request in automated driving, participants reported that they experienced slow cognitive processing and a feeling of numbness while at the same time, they experienced stress due to the rapid need for action in taking over vehicle control^42^. None of these studies, however, investigated the correlation between cortical, self-reported, and physiological arousal systematically. As indicated by a recent review article, there is insufficient research that relates physiological parameters with self-reported sleep inertia within the same participants^24^.

For the presented analysis, data from a driving simulator study was taken^43^. The study initially compared the effects of different napping durations and the impact of the sleep architecture on post-sleep takeover and driving behavior. The results showed that the nap duration had only a minor influence on post-sleep driving. In contrast, the characteristics of sleep depth and, in particular, the last sleep stage were significant predictors: sleepiness on the Karolinska Sleepiness Scale (KSS)^44^ was significantly increased after waking up from all NREM stages. Drivers made more driving errors after waking up from N2 and N3. Takeover times were particularly increased after N1, while only a non-significant trend was observed after N2 and N3. The baseline for all comparisons was that the drivers were already awake before the waking stimulus. The present analysis focuses on the process of awakening and aims to compare the cortical, subjective, and physiological response to a high-stress scenario, namely the takeover of vehicle control during automated driving, after sleep. We hypothesized that after sleep, low-frequency cortical activity is increased and high-frequency activity is decreased. Further, we hypothesized an increase in heart rate as an indicator of stress. The effects were expected to be more pronounced the deeper the participants had slept before waking up.

## Methods

The description of the sample, apparatus, measures, and procedure follows the methods described in our related previous publication^43^.

### Sample

Twenty-four volunteers participated in the driving simulator study. The participants were recruited from the participant panel of the Würzburg Institute for Traffic Sciences (WIVW). Participants’ age and gender were measured by self-report. The sample consisted of 10 women and 14 men, with no participants belonging to a diverse gender identity. The mean age was 37.5 years (SD = 13.3, Min = 22, Max = 66). Prior to the experiment, all participants had completed a driving simulator training to minimize simulator sickness^45^. All participants were informed about the purpose and procedure of the study, and that they could withdraw from participation at any moment of the experiment without a reason. Informed consent was obtained from all participants. Participants were paid a taxi to get to the research lab and back home because they were invited under partial sleep deprivation. The study was conducted in accordance with the Declaration of Helsinki^46^. The study was approved by the internal ethics officer of the WIVW as well as an independent ethics officer of the RUMBA project (Approval No. V596; dated 16 January 2023).

### Apparatus and driving task

The study was conducted in a high-fidelity driving simulator of the WIVW. For simulation and data logging, we used the software SILAB^®^ 7.0 (WIVW GmbH).

**Fig. 1:**
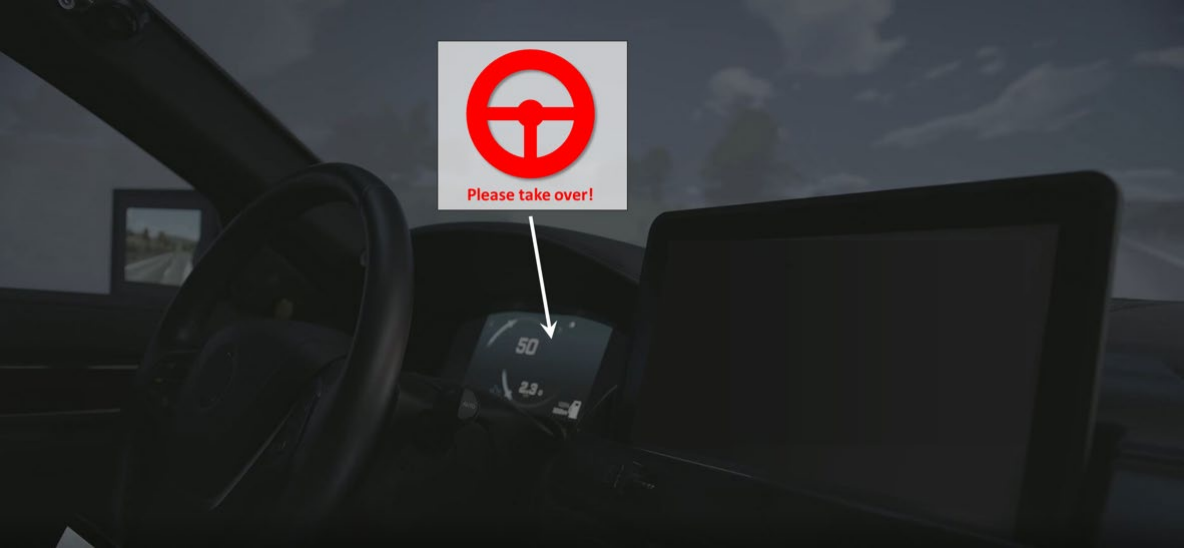
Cockpit of the high-fidelity driving simulator and visual takeover request which appeared in the cluster display as indicated by the white arrow (photo provided by WIVW, used with permission).

The simulated vehicle was equipped with an automated driving system. The automation was initiated by pressing a button on the steering wheel causing the backrest to automatically recline to a 45° lying position^47^. The automation then executed full vehicle guidance and the participants were temporarily relieved from all responsibilities concerning the driving task. Participants were informed by a voice prompt as to whether they were permitted to sleep during the automated drive. The participants were informed that the system might request them to resume control of the vehicle. In takeover scenarios, the system automatically adjusted the backrest to an upright position and issued a visual and acoustic takeover request (TOR). During the TOR, a red steering wheel symbol accompanied by the text “Please take over!” appeared on the cluster display (for an illustration, see Fig. 1). Simultaneously, a warning tone was emitted and an automatic voice output said “Please take over the driving task promptly. The automation will be deactivated in 30 seconds”. The warning tone then sounded once every 5 s. From 25 s after the start of the TOR, the warning tone sounded continuously for 5 s. After a total of 30 s, a continuous alarm signal sounded and the vehicle came to a standstill. The participants were asked to assume manual driving control as soon as possible and within a maximum time of 30 s by pressing the steering wheel button.

During the takeover, the vehicle was traveling along a straight rural road with no other traffic. After taking over, participants proceeded manually on an urban road. The manual drive took approximately five to ten minutes, depending on the participants’ driving speed. In the driving simulator, illumination and ambient noise were held constant between conditions.

### Measures

Heart rate was measured with a Polar T34 heart rate sensor (Polar Electro GmbH) and recorded with SILAB^®^ 7.0. The sample rate was 100 Hz.

Electroencephalogram (EEG) and electrooculogram (EOG) were recorded with active actiCAP-electrodes, the V-AMP amplifier, and the BrainVision Recorder software (all Brain Products GmbH). Six electrodes of 32 electrodes (F3, F4, C3, C4, O1, and O2) were placed according to the international 10–20-system^22^. The sample rate was 500 Hz.

Self-reported sleepiness was measured with the Karolinska Sleepiness Scale (KSS)^44^ ranging from 1 (extremely alert) to 9 (very sleepy, great effort to keep awake).

Measures of takeover and driving behavior were obtained and reported in our related previous publication^43^.

### Experimental design

**Fig. 2:**
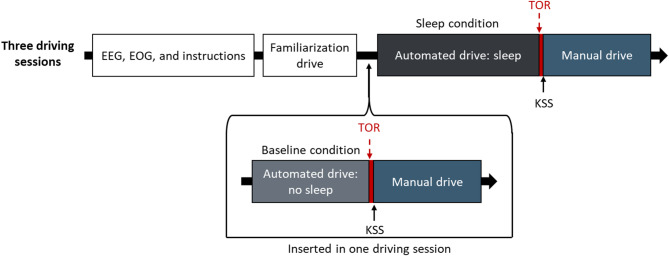
Schematic procedure of the experimental sessions. One of the three sessions included an additional block with automated and manual drive whose data serves as a baseline.

The experiment employed a repeated-measures design with five experimental conditions of which four are reported here. Participants experienced these four conditions across three sessions on different days in a balanced order. The experimental design and the procedure are illustrated in Fig. 2. In all three sessions, the participants were instructed to sleep during an automated drive and to drive manually after a TOR. For the analyses in our previous paper^43^ the duration of the nap opportunity was used as experimental condition. Therefore, the automated driving sections with sleep opportunities lasted 20, 40, and 60 min.

A baseline condition under wakefulness was added to one of the three sessions and presented before the respective sleep condition. Similar to the experimental conditions, it consisted of an automated driving section, a TOR, and a manual driving section. In the baseline condition, the participants listened to a podcast during the automated driving section to prevent sleep. In the baseline condition, the automated drive lasted 5 min. We did not balance the order of the baseline and sleep drive, as sleepiness (baseline) is a natural predecessor and sleep inertia is a natural successor of sleep.

For the analyses in this paper, the observed last sleep stage before the TOR served as a predictor, comparable to a quasi-experimental independent variable. It comprises the three EEG-coded sleep stages “W”, “N1”, and “N2 or N3”^18^. Details about the sleep stages are given in the introduction. Post-sleep heart rate, self-reported sleepiness, and cortical EEG-activity of the four frequency bands delta, theta, alpha, and beta served as dependent measures for arousal under sleep inertia. The independent and dependent measures are described in more detail in Sect. 2.6.

The sessions were conducted in the evening between 6 p.m. and midnight. The session start time was held constant for each participant. To promote sleep during the experiment, participants were instructed to sleep no more than four hours the night before participation, to avoid naps on the day of the experiment, and to refrain from consuming caffeine for at least four hours prior. Compliance with these instructions was verified through a questionnaire at the beginning of the experimental session. All participants adhered to the rules regarding caffeine intake and daytime napping. The average sleep duration the night before participation was 242.8 min (SD = 53.8, Min = 40.0, Max = 390). However, the individual sleep durations were comparable across the three experimental sessions as can be reviewed in Tomzig, et al.^43^.

### Procedure

Figure 2 illustrates the procedure of the experimental sessions. At the beginning of each session, the experimenter attached the EEG and EOG electrodes to the participant’s head. After being seated in the driving simulator, participants received a detailed explanation of the automated driving system and the manual driving task. To reduce first-contact effects, all participants completed a brief familiarization drive before each driving session, which included a short automated drive, a TOR, and a manual drive.

The experimental drive commenced with a brief manual drive on a rural road before the automated driving system became available. Participants were then asked to activate the automated driving system and to either sleep or stay awake, depending on the experimental condition as described in Sect. 2.4. After five to 60 min, depending on the experimental condition, the automated drive was interrupted by a TOR. Following the takeover, participants indicated their level of sleepiness on the KSS and completed the manual drive (for more details, see Tomzig, et al.^43^). In one of the three sessions, participants were not allowed to sleep in the first automated drive but underwent a second automated drive in which they were asked to sleep which was again terminated by a TOR. One session lasted approximately 90 to 150 min, depending on the experimental condition.

### Data processing and analysis

EEG signals were high-pass filtered at 0.5 Hz with a time constant of 0.3 s, low-pass filtered at 35 Hz, and notch filtered at 50 Hz. The sleep stages were visually coded by an expert following the American Academy of Sleep Medicine standard^18^. The EEG episodes were divided into epochs of 30 s each, and each epoch was assigned a distinct sleep stage.

For the spectral analyses, EEG data were preprocessed with BrainVision Analyzer 2.1 (Brain Products GmbH). Artifacts (e.g., due to signal noise or motion) were marked automatically by the software based on the following criteria: gradient > 50 µV/ms, absolute difference > 200 µV within 200 ms, amplitude < −100 µV or > 100 µV, maximum activity < 0.5 µV within 200 ms. Data 200 ms before to 200 ms after the detected event were coded as artifact. Detected artifacts were excluded from data analysis except if the data were previously coded as K-complex or as N3 (manual post-coding). Data were corrected against eye movements via independent component analysis (meaned slope algorithm and 30% of total value to delete based on the sum of squared corrections). Data were down-sampled to 256 Hz and segmented into intervals of 2 s length. Spectral analysis (Fast Fourier Transformation) was conducted, applying a 10% Hanning Window. Power spectral densities (PSD) of the four frequency bands delta (0.5 to 4.0 Hz), theta (4.0 to 8.0 Hz), alpha (8.0 to 12.0 Hz), and beta (12.0 to 35.0 Hz)^8^ were computed. Extreme outliers were winsorized by a factor of three times the interquartile range. Fifteen segments, each lasting 2 s, were averaged into larger segments, each spanning 30 s. As sleep inertia manifests stronger in posterior scalp locations^10^ the two occipital electrodes O1 and O2 were used for statistical data analysis. To obtain a single value per frequency band, data from both occipital electrodes were averaged.

We applied multilevel analyses^48^ (i.e., multiple regression models for repeated measures designs) to assess the influence of the last sleep stage on the PSD of the four frequency bands delta, theta, alpha, and beta as well as heart rate. The last sleep stage is an ordinal-scaled predictor with the three stages “W”, “N1”, and “N2 or N3” and indicates the sleep depth in the 30 s before the TOR was issued. N2 and N3 were combined for the analyses because participants were awakened from N3 in only three instances, and both N2 and N3 have been found to be followed by sleep inertia^49–51^. The significance of the stages “N1” and “N2 or N3” is tested against the category “W” as a reference. The designation “W” as the final sleep stage means that the participants were awake at the time the TOR was issued, for example, due to having awakened spontaneously or because they had failed to fall asleep.

To assess how long the last sleep stage affects heart rate and PSD, one multilevel analysis was conducted for each segment, starting with the takeover period, followed by segments of 30 s length representing the manual drive. There is comprehensive evidence that sleep inertia is strongest immediately after awakening and dissipates with time awake^1,2,13^. To avoid alpha inflation, we only tested until a non-significant result emerged after takeover. By means of visual data inspection and using Levene tests, all computed regression models were tested on homoscedasticity, which was given for all models (all *p* >.05). Further, there were no indications for influential cases (all Cook’s Distances < 1)^52^. The assumption of normality was tested by visual inspection and with Shapiro Wilk tests. In several models, there were indications for non-normal distributed residuals. Therefore, as a more robust approach, we applied bootstrapping with 2000 repetitions to compute the confidence intervals and p-values of the regression weights^52^.

We conducted repeated measures correlations^53^ to quantify the relations of heart rate, self-reported sleepiness (KSS), as well as delta, theta, alpha, and beta activity. Sleep inertia and, therefore, its EEG signature is expected to be highest immediately after awakening^11,13,54^ which is why we included the PSDs during takeover in the repeated measures correlation. In contrast, compared to brain activity, mean heart rate is an inert measure. Previous studies showed that heart rate increases in response to arousing stimuli^31,55,56^ and forced awakenings^41,57,58^. However, in forced awakenings, the peak of heart rate is commonly reached only after some tens of seconds^56–58^. To capture the expected increase in heart rate, we included the heart rate during the first 30 s after takeover in the repeated measures correlations instead of the heart rate during takeover.

Significance level was set at *p* <.05 if not indicated differently. For the repeated measures correlations, p-values were adjusted according to Bonferroni-Holm^59^. In this case, results were considered significant if the adjusted p-value *p*_*adj*_ was < 0.05. Results were considered as tendency if *p* <.05 but *p*_*adj*_ > 0.05.

We conducted the statistical analyses using the software R version 4.1.2^60^ with the packages rmcorr^53^ and lme4^61^. Due to the limited quantity of data, we employed restricted maximum likelihood estimation instead of maximum likelihood estimation^62^. The model tests and F-values were computed and corrected using a Kenward-Roger adjustment for small samples^62,63^.

## Results

**Fig. 3:**
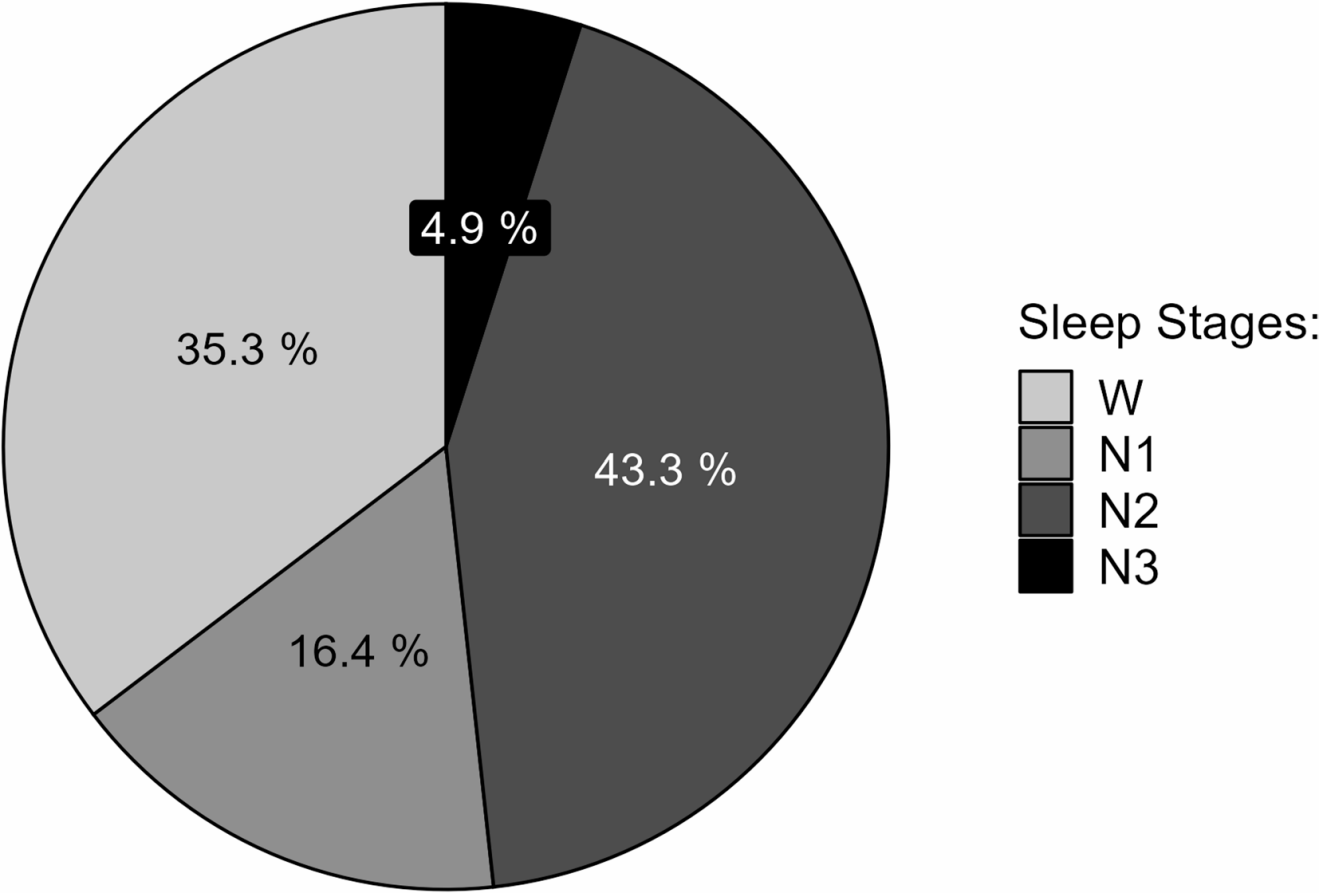
Mean distribution of sleep stages across all participants.

Across the three the sleeping conditions, mean sleep efficiency was 64.7% (i.e., the total sleep time by the duration of the napping opportunity). Figure 3 provides a breakdown across sleep stages. All 24 participants were awakened from EEG-verified sleep by the TOR in at least one driving session. In ten sessions, participants were awakened from N1, in 34 sessions from N2, and in three sessions from N3. In the 24 baseline drives without sleep opportunity and in 20 drives with sleep opportunity, participants were awake in the 30 s prior to the TOR. No participant was awakened from REM. In five datasets from different participants, the last sleep stage could not be coded due to signal noise. PSD during takeover could not be analyzed in 13 datasets and PSD after takeover could not be analyzed in four datasets, both due to artifacts or signal noise. Due to the varying amount of data, we indicate the included number of datasets with every statistical test.

**Fig. 4:**
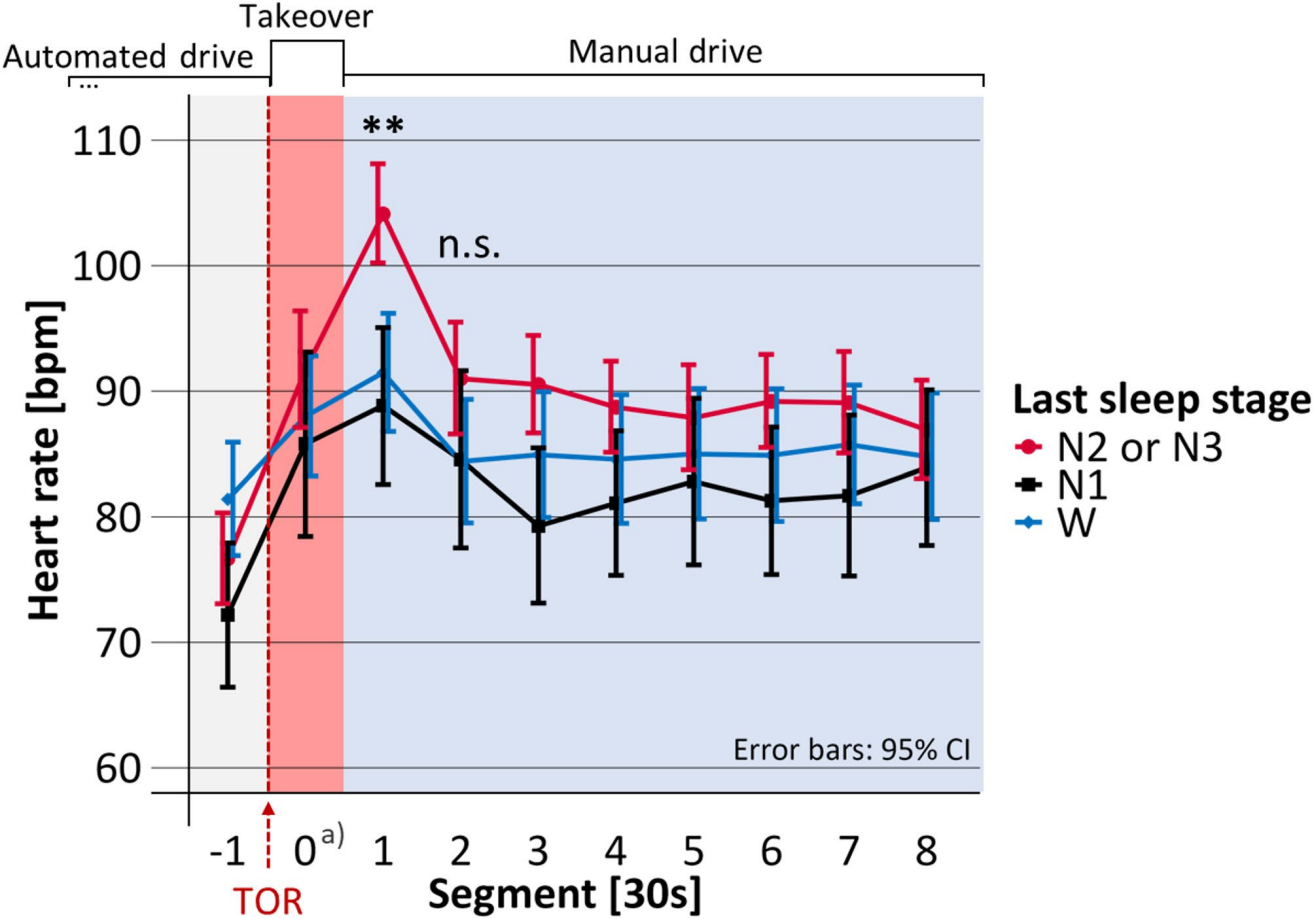
Mean heart rate before, during and after the takeover depending on the last sleep stage. TOR: Takeover request; bpm: beats per minute; Significance of regression model: *p <.05; **p <.01; ***p <.001; n.s. = not significant. a) Segment 0 shorter than 30 s.

**Fig. 5:**
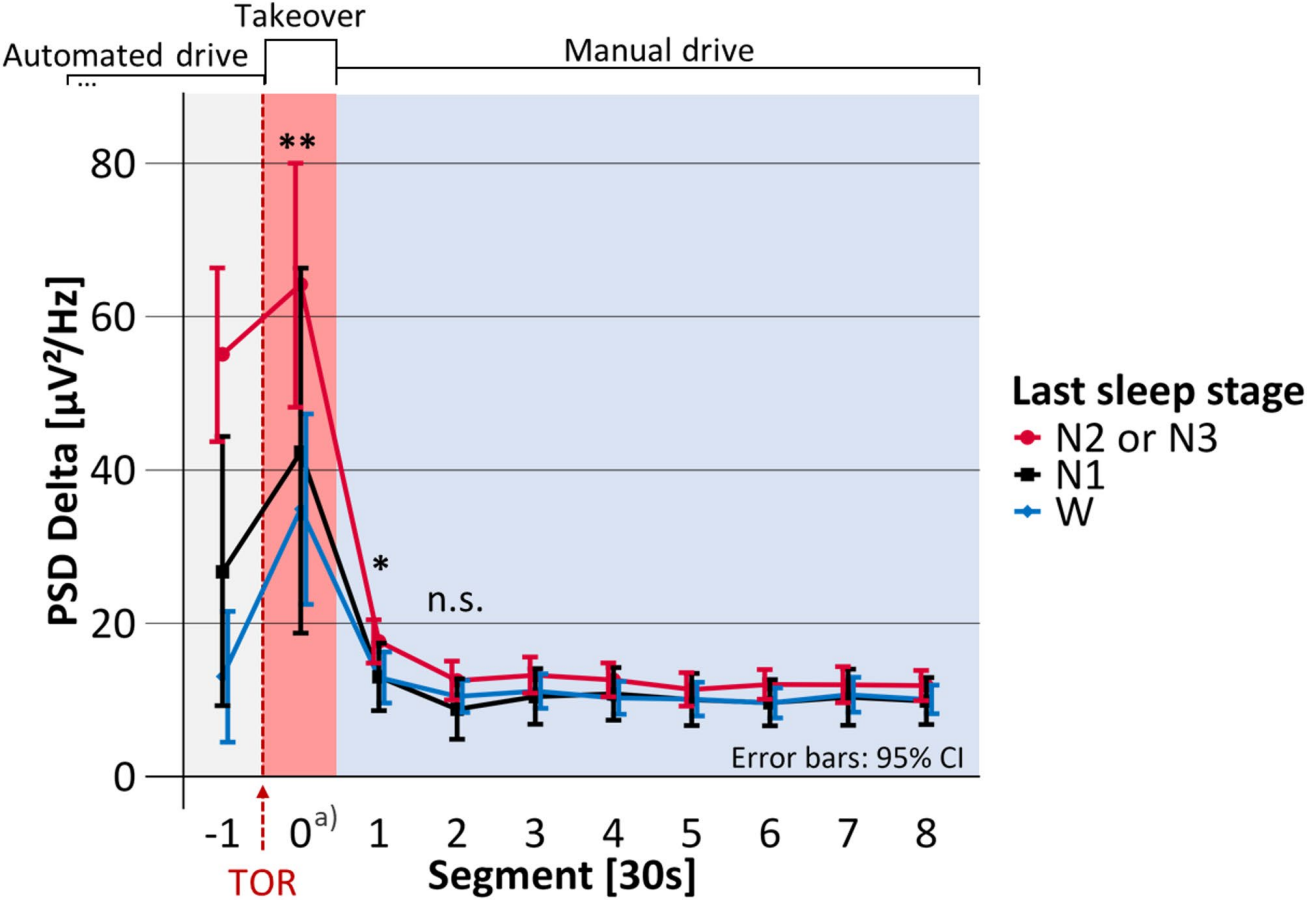
Mean occipital EEG delta activity before, during and after the takeover depending on the last sleep stage. PSD: Power spectral density; TOR: Takeover request; Significance of regression model: *p <.05; **p <.01; ***p <.001; n.s. = not significant. a) Segment 0 shorter than 30 s.

**Fig. 6:**
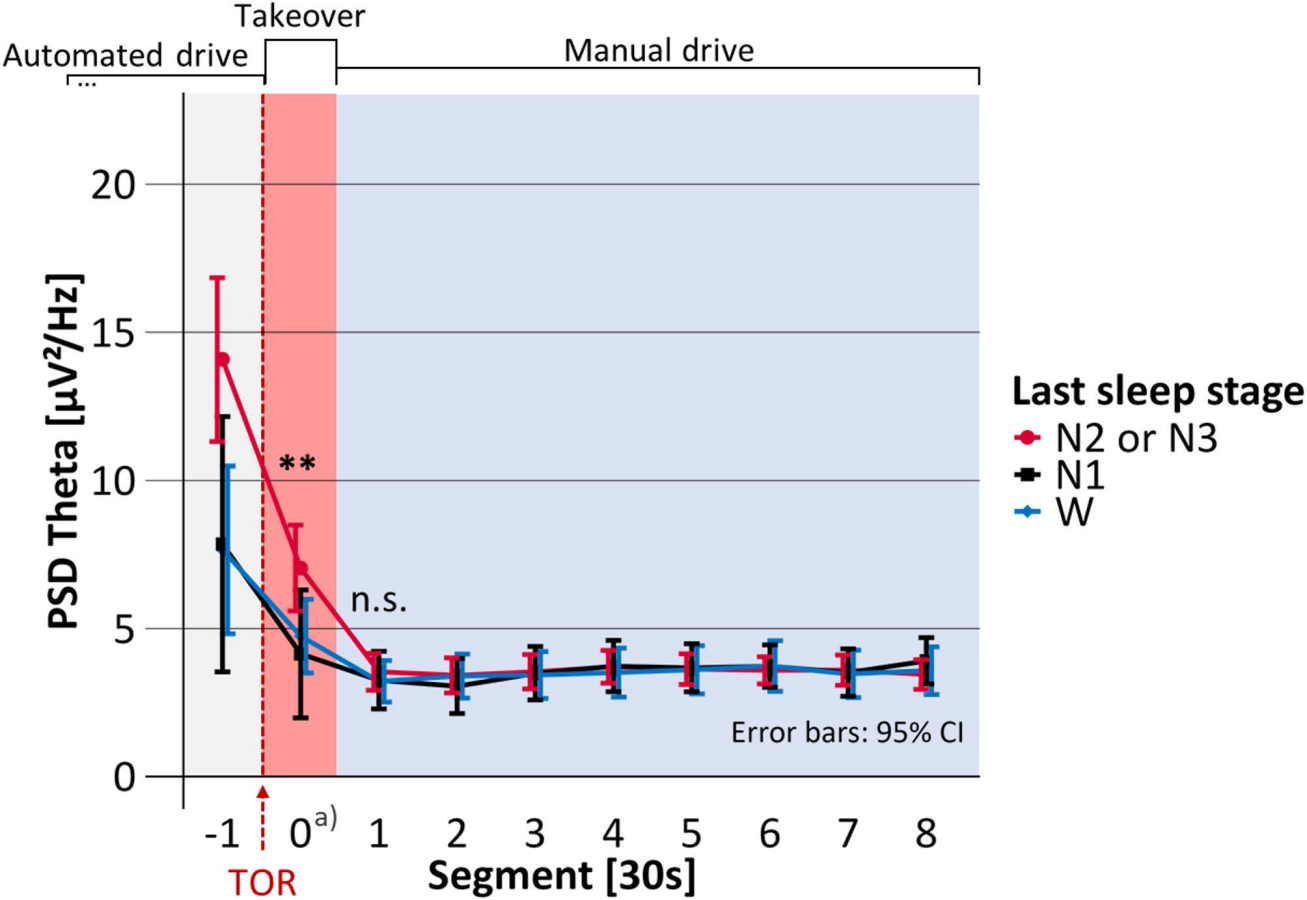
Mean occipital EEG theta activity before, during and after the takeover depending on the last sleep stage. PSD: Power spectral density; TOR: Takeover request; Significance of regression model: *p <.05; **p <.01; ***p <.001; n.s. = not significant. a) Segment 0 shorter than 30 s.

**Fig. 7:**
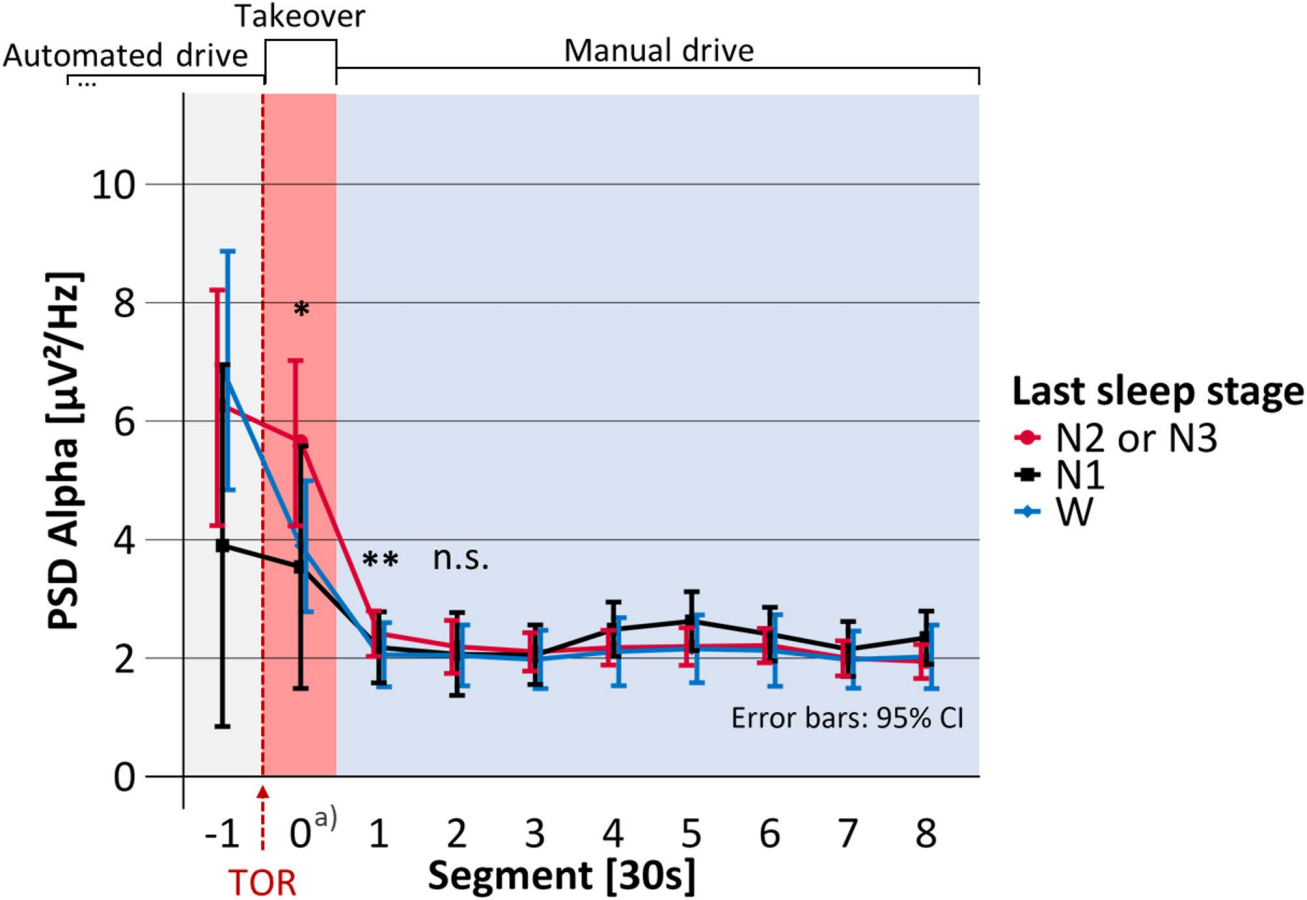
Mean occipital EEG alpha activity before, during and after the takeover depending on the last sleep stage. PSD: Power spectral density; TOR: Takeover request; Significance of regression model: *p <.05; **p <.01; ***p <.001; n.s. = not significant. a) Segment 0 shorter than 30 s.

**Fig. 8:**
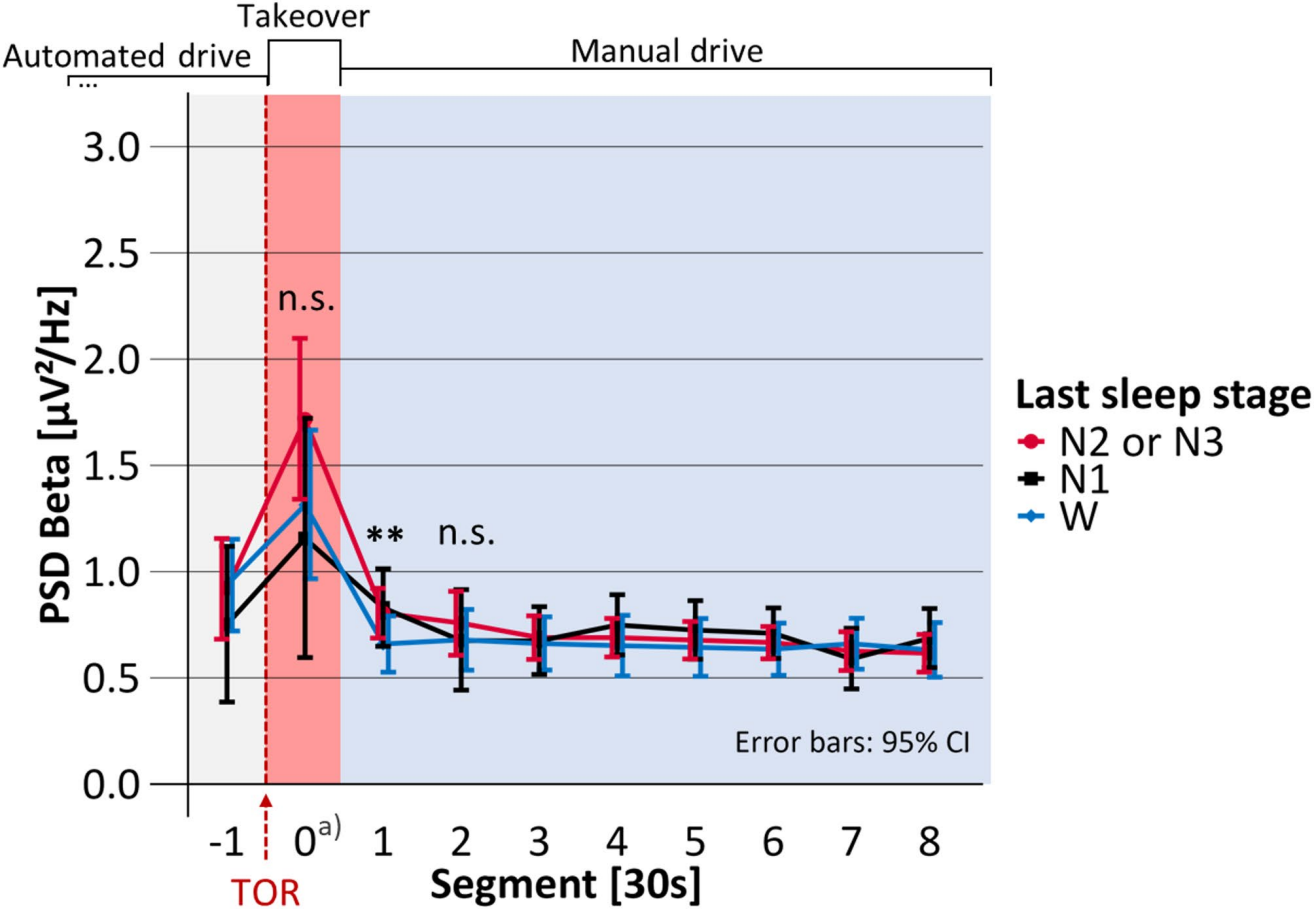
Mean occipital EEG beta activity before, during and after the takeover depending on the last sleep stage. PSD: Power spectral density; TOR: Takeover request; Significance of regression model: *p <.05; **p <.01; ***p <.001; n.s. = not significant. a) Segment 0 shorter than 30 s.

Mean takeover time, i.e., the time span between the start of the TOR until the participants successfully deactivated the automated driving system was 16.4 s (SD = 6.4). The duration of Segment 0 in Figs. 4, 5, 6, 7 and 8 is equal to the individual takeover time and is therefore generally shorter than the duration of the other segments. Descriptive data for the obtained measures is summarized in Table 1.

**Table 1:** Means and SD for heart rate (HR) and power spectral densities of occipital delta, theta, alpha, and beta activity.

		Segment 0					Segment 1					Segment 2				
Last sleep stage	Statistic	HR	Delta	Theta	Alpha	Beta	HR	Delta	Theta	Alpha	Beta	HR	Delta	Theta	Alpha	Beta
W	Mean	87.99	34.92	4.75	3.90	1.31	91.47	12.93	3.22	2.06	0.66	84.39	10.46	3.39	2.05	0.68
	SD	13.67	26.13	3.09	2.71	0.96	12.97	8.36	1.79	1.24	0.34	13.39	5.60	1.89	1.14	0.38
N1	Mean	85.80	42.29	4.14	3.54	1.16	88.85	13.02	3.25	2.18	0.83	84.54	8.79	3.05	2.06	0.68
	SD	13.06	42.01	2.29	1.74	0.50	12.13	8.03	1.64	1.17	0.43	17.07	5.48	1.79	1.10	0.29
N2 or N3	Mean	91.69	64.19	7.04	5.65	1.72	104.12	17.63	3.54	2.42	0.81	90.99	12.53	3.42	2.19	0.76
	SD	12.66	43.21	4.08	3.85	1.01	12.05	9.61	1.98	1.50	0.34	12.33	6.92	1.99	1.59	0.43

*Note. *Means and SD for HR in beats per minute (bpm). Means and SD for delta, theta, alpha and beta activity in µV²/Hz.

The results on the effect of the last sleep stage on self-reported sleepiness are not reported here, as they have already been published in another paper^43^.

### Heart rate

As shown in Fig. 4 the maximum of the average heart rate was not reached during the takeover (Segment 0), but only later, in the first 30 s after takeover (in Segment 1). In Segment 1, the heart rate differed significantly depending on the last sleep stage (F(2, 71.1) = 12.53, *p* <.001, marginal R² = 0.126, *N* = 91). The model predicted that heart rate was not significantly increased after awakening from N1, compared to W, but that it was significantly increased when the participants had been awakened from N2 or N3, compared to W (see Table 2; Fig. 4). After waking from N2 or N3, the model predicted the heart rate to be increased by 9.52 beats per minute compared to W. After awakenings from N2 or N3, the heart rate remained elevated throughout the first four minutes of the manual drive. However, in the period 30 to 60 s after takeover (i.e., Segment 2 in Fig. 4), the last sleep stage did not significantly predict heart rate (F(2, 72.3) = 1.86, *p* =.163, marginal R² = 0.024, *N* = 91) and statistical testing was terminated.

**Table 2:** Regression results regarding the association between the last sleep stage and heart rate in Bpm after the takeover request.

	Segment 1					Segment 2				
Predictor	b	95% CI		β	*p*	b	95% CI		β	*P*
		LL	UL				LL	UL		
Intercept	92.68	88.20	97.40	−0.26	< 0.001	85.87	81.18	90.70	−0.09	< 0.001
Last sleep stage = N1	−1.26	−7.42	5.04	−0.09	0.692	−1.94	−8.79	5.02	−0.14	0.612
Last sleep stage = N2 or N3	**9.52**	**5.56**	**13.40**	**0.68**	**< 0.001**	3.78	−0.69	8.23	0.28	0.093

*Note. b* represents unstandardized regression weights. *95% CI*: 95% confidence interval of *b*. *LL* and *UL* indicate the lower and upper limits of a confidence interval, respectively. β represents the standardized regression weights. Significant effects are printed in bold.

### Delta activity

Statistical results on the occipital delta activity are displayed in Table 3; Fig. 5. The last sleep stage significantly predicted the delta activity during Segment 0 (F(2, 70.8) = 7.10, *p* =.002, marginal R² = 0.143, *N* = 80). According to the model, delta activity did not significantly differ after awakening from N1, compared to W. However, after waking up from N2 or N3, the model predicted the delta activity to be significantly increased by 30.64 µV²/Hz, compared to W. In the 30 s after the takeover (Segment 1), delta activity still significantly depended on the last sleep stage (F(2, 69.0) = 4.59, *p* =.013, marginal R² = 0.052, *N* = 88). According to the model, delta activity was not significantly increased after awakenings from N1 but it was significantly increased after N2 or N3 by 4.37 µV²/Hz, both compared to W. In the period 30 to 60 s after takeover (i.e., Segment 2 in Fig. 5), the last sleep stage did not significantly predict the delta activity (F(2, 77.4) = 2.18, *p* =.120, marginal R² = 0.043, *N* = 88) and testing was terminated.

**Table 3:** Regression results regarding the association between the last sleep stage and occipital delta activity in µV²/Hz after the takeover request.

	Segment 0					Segment 1					Segment 2				
Predictor	b	95% CI		β	*p*	b	95% CI		β	*p*	b	95% CI		β	*p*
		LL	UL				LL	UL				LL	UL		
Intercept	34.35	21.71	46.77	−0.35	< 0.001	12.77	9.44	15.95	−0.23	< 0.001	10.50	8.38	12.50	−0.10	< 0.001
Last sleep stage = N1	4.75	−19.07	28.49	0.12	0.690	0.96	−3.51	5.48	0.11	0.662	−2.15	−6.18	1.89	−0.35	0.287
Last sleep stage = N2 or N3	**30.64**	**14.29**	**46.63**	**0.81**	**0.002**	**4.37**	**1.56**	**7.24**	**0.48**	**0.004**	1.91	−0.61	4.47	0.31	0.164

*Note. b* represents unstandardized regression weights. *95% CI*: 95% confidence interval of *b*. *LL* and *UL* indicate the lower and upper limits of a confidence interval, respectively. β represents the standardized regression weights. Significant effects are printed in bold.

### Theta activity

Statistical results on the occipital theta activity are displayed in Table 4; Fig. 6. The last sleep stage significantly predicted the theta activity during Segment 0 (F(2, 67.4) = 5.54, *p* =.006, marginal R² = 0.106, *N* = 80). According to the regression model, theta activity did not significantly differ after awakening from N1, compared to W. However, after awakening from N2 or N3, theta activity was predicted to be significantly increased by 2.29 µV²/Hz, compared to W. After takeover, theta activity was not significantly predicted by the last sleep stage (F(2, 69.7) = 1.08, *p* =.345, marginal R² = 0.014, *N* = 88). Therefore, testing was stopped.

**Table 4:** Regression results regarding the association between the last sleep stage and occipital theta activity in µV²/Hz after the takeover request.

	Segment 0					Segment 1				
Predictor	b	95% CI		β	*p*	b	95% CI		β	*p*
		LL	UL				LL	UL		
Intercept	4.74	3.49	6.00	−0.24	< 0.001	3.24	2.55	3.94	−0.06	< 0.001
Last sleep stage = N1	−0.53	−2.67	1.56	−0.15	0.643	−0.29	−1.26	0.69	−0.16	0.554
Last sleep stage = N2 or N3	**2.29**	**0.86**	**3.80**	**0.63**	**0.006**	0.37	−0.26	1.00	0.20	0.272

*Note. b* represents unstandardized regression weights. *95% CI*: 95% confidence interval of *b*. *LL* and *UL* indicate the lower and upper limits of a confidence interval, respectively. β represents the standardized regression weights. Significant effects are printed in bold.

### Alpha activity

Statistical results on the occipital alpha activity are displayed in Table 5; Fig. 7. The last sleep stage significantly predicted the alpha activity during Segment 0 (F(2, 69.7) = 4.78, *p* =.011, marginal R² = 0.099, *N* = 80). According to the regression model, alpha activity did not significantly differ after awakening from N1, compared to W. However, after waking up from N2 or N3, alpha activity was predicted to be significantly increased by 2.08 µV²/Hz, compared to W. Nevertheless, in the 30 s after the takeover (i.e., Segment 1), alpha activity still significantly depended on the last sleep stage (F(2, 67.1) = 5.16, *p* =.008, marginal R² = 0.045, *N* = 88). Alpha activity was predicted not to be significantly increased after awakenings from N1, but to be significantly increased after N2 or N3 by 0.58 µV²/Hz, both compared to W. In the period 30 to 60 s after takeover (i.e., Segment 2), the last sleep stage did not significantly predict the alpha activity (F(2, 69.4) = 1.95, *p* =.150, marginal R² = 0.025, *N* = 88) and testing was terminated.

**Table 5:** Regression results regarding the association between the last sleep stage and occipital alpha activity in µV²/Hz after the takeover request.

	Segment 0					Segment 1					Segment 2				
Predictor	b	95% CI		β	*p*	b	95% CI		β	*p*	b	95% CI		β	*p*
		LL	UL				LL	UL				LL	UL		
Intercept	3.73	2.66	4.85	−0.26	< 0.001	2.02	1.48	2.56	−0.15	< 0.001	2.02	1.52	2.54	−0.06	< 0.001
Last sleep stage = N1	−0.18	−2.23	1.80	−0.06	0.867	−0.14	−0.74	0.46	−0.10	0.644	−0.32	−1.02	0.37	−0.25	0.375
Last sleep stage = N2 or N3	**2.08**	**0.70**	**2.96**	**0.65**	**0.006**	**0.58**	**0.20**	**0.97**	**0.44**	**0.007**	0.34	−0.11	0.79	0.26	0.141

*Note. b* represents unstandardized regression weights. *95% CI*: 95% confidence interval of *b*. *LL* and *UL* indicate the lower and upper limits of a confidence interval, respectively. β represents the standardized regression weights. Significant effects are printed in bold.

### Beta activity

Statistical results on the occipital beta activity are displayed in Table 6; Fig. 8. The last sleep stage did not significantly predict the beta activity during Segment 0 (F(2, 65.1) = 2.10, *p* =.130, marginal R² = 0.039, *N* = 80). After takeover, beta activity was significantly predicted by the last sleep stage (F(2, 69.6) = 5.03, *p* =.009, marginal R² = 0.060, *N* = 88). The model predicted that beta activity was not significantly increased after awakenings from N1 but significantly increased after N2 or N3 by 0.19 µV²/Hz, both compared to W. In the period 30 to 60 s after takeover (i.e., Segment 2), the last sleep stage did not significantly predict the beta activity (F(2, 73.1) = 1.58, *p* =.213, marginal R² = 0.027, *N* = 88) and testing was terminated.

**Table 6:** Regression results regarding the association between the last sleep stage and occipital beta activity in µV²/Hz after the takeover request.

	Segment 0					Segment 1					Segment 2				
Predictor	b	95% CI		β	*p*	b	95% CI		β	*p*	b	95% CI		β	*p*
		LL	UL				LL	UL				LL	UL		
Intercept	1.36	1.01	1.71	−0.10	< 0.001	0.65	0.52	0.78	−0.25	< 0.001	0.68	0.54	0.83	−0.07	< 0.001
Last sleep stage = N1	−0.21	−0.77	0.36	−0.22	0.486	0.12	−0.07	0.30	0.33	0.229	−0.11	−0.34	0.13	−0.27	0.390
Last sleep stage = N2 or N3	0.33	−0.06	0.71	0.34	0.109	**0.19**	**0.07**	**0.30**	**0.53**	**0.006**	0.10	−0.05	0.24	0.25	0.207

*Note. b* represents unstandardized regression weights. *95% CI*: 95% confidence interval of *b*. *LL* and *UL* indicate the lower and upper limits of a confidence interval, respectively. β represents the standardized regression weights. Significant effects are printed in bold.

### Correlations between EEG-activity, self-reported sleepiness, and heart rate

Self-reported sleepiness increased significantly with occipital delta and alpha activity and tendentially with theta activity during takeover (see Table 7). The correlation between beta activity and self-reported sleepiness was not significant. The occipital delta activity during takeover also significantly predicted the heart rate after takeover which significantly increased with the PSD of delta waves. Theta, alpha, and beta activity did not significantly predict the post-takeover heart rate. A higher heart rate was significantly associated with higher levels of self-reported sleepiness, as displayed in Table 7.

**Table 7:** Repeated measures correlations between cortical delta and theta activity during takeover, self-reported sleepiness, and heart rate after takeover.

Measure 1	Measure 2	*r*_rm_	*N*	df	*p*	*p*_adj_
**PSD Delta** ^1)^	**Sleepiness**	**0.481**	**83**	**59**	**< 0.001**	**< 0.001**
PSD Theta^1)^	Sleepiness	0.314	83	59	0.014	0.067
**PSD Alpha** ^1)^	**Sleepiness**	**0.335**	**83**	**59**	**0.008**	**0.049**
PSD Beta^1)^	Sleepiness	0.187	83	59	0.149	0.298
**PSD Delta** ^1)^	**Heart rate** ^2)^	**0.380**	**83**	**59**	**0.003**	**0.018**
PSD Theta^1)^	Heart rate^2)^	0.252	83	59	0.050	0.201
PSD Alpha^1)^	Heart rate^2)^	0.222	83	59	0.085	0.256
PSD Beta^1)^	Heart rate^2)^	0.058	83	59	0.658	0.658
**Sleepiness**	**Heart rate** ^2)^	**0.358**	**96**	**71**	**0.002**	**0.015**

Note. PSD: Power spectral density; 1) during takeover (Segment 0); 2) first 30 s after takeover (Segment 1). Significant effects are printed in bold.

## Discussion

In the presented study, we analyzed the cortical, subjective, and physiological response to a high-stress scenario after sleep. We compared these responses depending on the last sleep stage prior to a takeover request in an automated driving scenario. After waking from N2 or N3, a paradoxical relationship between cortical, self-reported, and physiological arousal became evident.

After awakening from N2 or N3, the occipital delta, theta, alpha, and beta activity as well as the heart rate were significantly increased compared to napping opportunities in which the participants had already been awake before the TOR. Occipital delta, theta, and alpha activities were increased during the takeover phase for up to 30 s. In the following 30 s of task execution (i.e., driving manually), heart rate and occipital delta, alpha, and beta activity were elevated. None of these measures was significantly increased after awakening from N1. In our related previously published analysis, the post-sleep self-reported sleepiness was increased not only after awakening from N2 or N3 but also after N1, compared to being awake before the TOR^43^. Increased self-reported sleepiness and increased activities in the low-frequency bands delta, theta, and alpha represent lower arousal, and increased high-frequency beta activity and increased heart rate both represent high arousal^4^.

The association between the last sleep stage and post-sleep physiological and cortical arousal indicates sleep inertia. Significant sleep inertia effects on physiological and cortical arousal occurred when the participants were awakened from N2 or N3 but not after N1. The increased low-frequency activity (delta, theta, and alpha) under sleep inertia supports findings from previous studies^10–12^. The activity of the low-frequency bands was significantly (delta and alpha) or tendentially (theta) correlated with increased self-reported sleepiness. Our results demonstrate that the cortical low-frequency activity is only increased after awakenings from N2 or N3 but not from N1 indicating the impact of the last sleep stage on cortical arousal upon awakening.

An elevation in heart rate during arousal events or upon waking is a typical physiological response^28,30,31^. The results of our study indicate that the heart rate level is higher after waking from N2 or N3 compared to W. There is a research gap regarding the dependence of cardiovascular activity on the last sleep stage. A study conducted with participants who awoke in a quiet environment revealed that heart rate was higher after waking from N2 than after waking from REM^64^. However, the study had a limited sample size and employed a between-subjects design, which did not compare the results to a pre-sleep state of sleepiness or to participants who had waked prematurely. Therefore, it is not possible to ascertain whether an elevated heart rate is a genuine indicator of sleep inertia or if it is triggered by other factors, such as stress, as discussed further below. In contrast, the chosen within-subjects design for this study allowed to control for interindividual differences in susceptibility to sleep inertia^65^ physiological reactivity to stimuli^66^ and sleep disorders^67^ which could potentially bias effects in the presented context. We carefully counterbalanced the session order to limit habituation effects^68^ which may have occurred due to repeated exposure to the experimental stimuli (e.g., the TOR)^68^.

Contrary to previous studies^10–12^ which examined the post-sleep EEG-activity in resting participants, in our study, the cortical beta activity was not reduced after awakening from N2 or N3 but increased. Altogether, the presented results seem paradoxical: The cortical delta, theta, and alpha-activity and the elevated self-reported sleepiness indicate low arousal after awakening from N2 or N3. The elevated heart rate and cortical beta activity both represent high arousal. A key feature of the paradox is that under sleep inertia, increased heart rate was significantly associated with increased delta activity and increased self-reported sleepiness. Some arousal theories and definitions view arousal as a holistic construct in which physiological, cortical, and self-reported measures should uniformly indicate low or high arousal^3,4^. Therefore, an increase in cortical arousal would be assumed to go along with an increase in physiological and self-reported arousal. Conversely, our findings indicate that increased delta activity and self-reported sleepiness (both representing low arousal) were associated with an elevated heart rate (representing high arousal).

This arousal paradox and the discrepant results compared to previous studies^10–12^ can be explained by the context of our study where participants were asked to perform a critical task immediately after awakening. Increased beta activity has been found to be associated with enhanced cortical arousal due to mental effort or stress^69^. Mental effort refers to the required resources that must be mobilized to carry out a certain task^70^. A paradoxical increased beta activity has also been found in sleep-deprived participants where increased beta activity may indicate efforts to stay awake^71^. The increase in heart rate and especially its relation with high delta power may represent a stress response of the awakening body^72^. The takeover of the driving task in the context of automated driving implies multiple cognitive sub-tasks which is highly demanding^73^. Previous research has demonstrated that cognitive processing is slowed down under sleep inertia^1,23^ and that individuals perceive their cognitive capabilities to be reduced. This cognitive deceleration may be explained by the increased low-frequency activity^1^. According to the Transactional Model of Stress and Coping^74^ a gap between capabilities and task demands represents a stressor^75^. We assume that the perceived mismatch between the high task demands of the takeover and the cognitive decrements have led to stress. Hence, our results possibly indicate the parallel presence of two processes:

1. Sleep inertia which is marked by increased low-frequency activity and increased self-reported sleepiness.
2. Enhanced mental effort and stress resulting from the mismatch between high task demands and slow cognitive processing due to sleep inertia

We consider mental effort a possible explanation for the increased high-frequency activity that may have been required to compensate cognitive decrements and solve the takeover task under a state of sleep inertia. The parallel presence of situationally reduced cognitive capabilities due to sleep inertia and high task demands may elicit stress as reflected by the increased heart rate. Given the statistical dependency on the last sleep stage, the physiological stress is not exclusively a reaction to the waking stimulus (i.e., the takeover request). Instead, under the presented conditions, it seems to be a consequence of sleep inertia. Future research should include measurements of mental effort, cognitive demands, and stress to validate this explanation.

We assume that this arousal paradox is not a universal characteristic of sleep inertia but only manifests in certain contexts, such as forced awakenings in which immediate cognitive performance is required. While the study was conducted in an automated driving scenario, the results might transfer to other domains where immediate action is required after awakening, such as shift work in hospitals, firefighters, power plants, or other domains of transport like aviation.

The observed effects of the last sleep stage on cortical arousal lasted shorter than expected. Those studies that did not include critical tasks immediately after awakening observed EEG patterns which are associated with sleep inertia over a period of five to ten minutes^10–12^. Stress might have counteracted sleep inertia. Under stress, catecholamines such as adrenaline and noradrenaline are released, triggering a flight-or-fight response^72^. This includes increased brain blood flow, accelerated breathing, and increased heart rate. Because of its activating effect, it is assumed that stress, mediated by adrenaline, could represent a countermeasure against sleep inertia^26,32^. However, our results cannot prove the absence of sleep inertia after 30 s of manual driving. Our analysis focused on occipital cortical brain activity because previous studies indicated this cortical area as most sensitive for sleep inertia^10,12^. However, sleep inertia can also affect subcortical regions, which can be assessed via cerebral blood flow^12,76^. Since EEG captures only cortical activity, it does not indicate whether sleep inertia persisted in subcortical areas. We assume that sleep inertia persisted because our previous analysis^43^ showed that participants made more driving errors after waking from N2 or N3 compared to W. Since the driving errors were recorded over several minutes following takeover, these findings cannot be accounted for by the arousal patterns presented in this paper.

Nonetheless, arousal may affect performance in safety-critical tasks^5^ and previous research has linked the observed behavioral changes under sleep inertia to low cortical arousal^1^. In various contexts, where it is important to be awake immediately after sleep, such as when individuals have to take over control in automated driving or in aviation, guidelines and strategies are being developed to ensure that the effects of sleep inertia on performance, comfort, and arousal are minimized^26,77–79^. In our initial study, it was an unexpected result that takeover times were particularly increased after awakening from N1 but not after N2 or N3^43^. In light of the present results, we assume that stress after N2 or N3 might have led to quicker, but potentially more hurried takeovers. Together, both analyses demonstrate that the last sleep stage should be considered when developing guidelines and wake-up concepts. After forced awakenings, countermeasures are needed to mitigate the effects of sleep inertia. Future research should investigate whether targeted stressors can be utilized as a reactive countermeasure. Besides performance, it is conceivable that arousal additionally influences well-being during the wake-up process. Over time, this could affect job satisfaction and user experience. It is a concern that a stress-inducing countermeasure may have a detrimental impact on well-being when waking up^80^. It is thus imperative that wake-up concepts for operational contexts carefully calibrate post-sleep arousal. Theories such as the Yerkes-Dodson-Law^5^ indicate that it might be beneficial to calibrate arousal to a medium level to enhance performance and well-being. Attempts have been made to increase cognitive arousal after waking while keeping physiological arousal low as a countermeasure, yet these have not yielded significant results^41^.

Furthermore, our results suggest that to achieve optimal arousal after sleep, individuals should not wake up from N2 or N3 (compared to N1). This may be realized by gradually waking a person. Even if it is not entirely clarified whether they have a significant impact on the last sleep stage^81^ dawn simulations prior to awakening have been proven to reduce detrimental effects of sleep inertia^81–83^. Further, in the presented study, the significant effects under sleep inertia diminished relatively rapidly. It is therefore recommended that individuals should be awakened timely before being asked to take over a task.

Our findings should be considered in light of certain limitations. To assess the effects of sleep inertia in a real-world context, research should be conducted in an ecologically valid setup. This setup, however, limited the standardization and controllability of factors as compared to sleep laboratories^10–12^. While we carefully removed motion artifacts from the data, the general increase of the delta- and beta activity during the takeover might be a residual artifact of either the takeover itself (e.g. warning sound) or the participants’ movements (see Figs. 5 and 8). We therefore did not interpret the absolute levels of EEG-activities but only the differences between the quasi-experimental conditions (i.e., the last sleep stages).

During the study design phase, the research questions in this paper were considered secondary. As a result, the study design was not fully optimized for these analyses. For example, as reported in our previous publication^43^ the takeover time was an important measure of takeover behavior under sleep inertia. However, this led to unequal takeover times and thus unequal lengths of Segment 0 in Figs. 4, 5, 6, 7 and 8. As the takeover times were also associated with the last sleep stage^43^ the results on the absolute duration of sleep inertia are slightly biased. The temporal effects should therefore only be interpreted on a functional level considering the underlying task (i.e., taking over manual driving vs. conducting the driving task).

The explanation that stress and mental effort led to the observed arousal paradox remains a hypothesis. The study design did not allow for a clear differentiation between sleep inertia and stress. Therefore, future studies should add a low-stress condition (e.g., awakening without a task requirement) as a baseline. Future studies should consider to apply further physiological measures, such as the systolic blood pressure which is more sensitive to mental effort than heart rate^84^. Moreover, while mental effort may lead to increased beta activity also in occipital electrode sites^85^ future studies should consider including frontal recordings as they may be more sensitive for cognitive workload^86^. Further, future studies should include suitable measures for takeover quality and relate them to cortical brain activity. The takeover times as measured in^43^ are not an exclusive performance indicator as this measure is largely influenced by motivational factors^87^.

The limited occurrence of awakenings from N3 prevented a separate analysis of the effects of N2 and N3. Given that N3 may have more severe effects on sleep inertia than N2^1^, our findings may overestimate the effects of N2 and underestimate the effects of N3. N3 typically begins approximately 30 min after sleep onset but may start earlier under conditions of high homeostatic sleep pressure^19^. Given the partial sleep deprivation in our participants, we had initially expected to observe more N3. We consider that the unfamiliar environment and semi-recumbent sleep position may have limited the occurrence of N3. In contrast, the absence of REM sleep was expectable, as REM typically occurs more frequently towards the end of the night^19^. The results are therefore limited to NREM sleep.

Regarding the effects, it should be considered that the wake condition represents a state of sleepiness. The effects of sleep inertia are expected to be even stronger when compared to a state of daytime alertness.

## Conclusion

The main finding of the study is that an arousal paradox may manifest under sleep inertia in high-demand conditions. The arousal paradox refers to the observed phenomenon that

1. Individuals experience low levels of subjective and cortical arousal,
2. Individuals simultaneously experience heightened physiological arousal, as well as indications of elevated mental effort,
3. That these dimensions are interrelated.

 Additionally to the requirement to assume a task, the conditions imply an urgency, sleeping in a reclined position and sitting up to react^88^ awakening in a moving environment, being immediately exposed to external stimuli, and finding oneself in a situation where one is typically fully alert. Such scenarios may arise for aircraft pilots^77^ rescue services^89^ and – with the advent of automated driving – drivers^90,91^. In such a situation, it can be presumed that sleep inertia increases stress. The low cortical and self-reported arousal which is characteristic of sleep inertia is thus accompanied by elevated arousal in other components. The arousal paradox under sleep inertia as observed in the presented study is not in line with arousal theories that consider arousal a holistic state of the human organism^3^. While further research is required, the results indicate that arousal may not be a homogeneous construct but rather involve distinct components, which can be influenced by various internal and external factors.

The presented study contributes to the refinement of theories and definitions of sleep inertia and arousal. Based on the results, the potential for the development of arousal-based countermeasures against sleep inertia in the future is indicated.

## Data Availability

The datasets analyzed during the current study are available from the corresponding author on reasonable request (contact: tomzig@wivw.de).
